# Use of network pharmacology and molecular docking to explore the mechanism of action of curcuma in the treatment of osteosarcoma

**DOI:** 10.1038/s41598-023-36687-z

**Published:** 2023-06-13

**Authors:** Minhua Hu, Hongsong Yan, Haishan Li, Yuanlan Feng, Weipeng Sun, Yueyi Ren, Luyao Ma, Wenxing Zeng, Feng Huang, Ziwei Jiang, Hang Dong

**Affiliations:** 1 The First Clinical College, Guangzhou University of Chinese Medicine, Guangzhou, China; 2 The First Affiliated Hospital of Guangzhou University of Chinese Medicine, Guangzhou, China

**Keywords:** Bone cancer, Pharmacology, Drug development

## Abstract

Curcuma has been used as an adjuvant treatment for osteosarcoma (OS) due to its anticancer compounds. However, the underlying mechanism remains unclear. Therefore, this study aimed to explore the mechanism of action of curcuma in the treatment of OS using network pharmacology and molecular docking. In this study, anticancer compounds were obtained from relevant literature, and curcuma-related targets and OS treatment targets were obtained from public databases. Protein‒protein interaction networks were constructed to screen out the hub genes using the STRING database and Cytoscape software. Cluster analysis of the protein modules was then performed using the Cytoscape MCODE plugin. Furthermore, Gene Ontology enrichment and Kyoto Encyclopedia of Genes and Genomes pathway analyses were performed for common targets among curcuma targets and OS-related targets using the DAVID database. Finally, molecular docking was performed, and the results were verified by Auto dock Tool and PyMOL software. Our research identified 11 potential active compounds, 141 potential therapeutic targets and 14 hub genes for curcuma. AKT1, TNF, STAT3, EGFR, and HSP90AA1 were the key targets closely related to the PI3K/Akt signaling pathways, HIF-1 signaling pathways, ErbB signaling pathways, and FOXO signaling pathways, which are involved in angiogenesis, cancer cell proliferation, metastasis, invasion, and chemotherapy resistance in the microenvironment of OS. Molecular docking suggested that the core compound had a strong affinity for key targets, with a binding energy of less than – 5 kJ/mol. The study showed that curcuma-mediated treatment of OS was a complex process involving multiple compounds, targets, and pathways. This study will enhance the understanding of how curcuma affects the proliferation and invasion of OS cells and reveal the potential molecular mechanism underlying the effect of curcuma on OS lung metastasis and chemotherapy resistance.

## Introduction

Osteosarcoma (OS) is one of the most common types of malignant bone tumors originating from bone tissue and is frequently diagnosed in adolescents, with most tumors appearing in the distal femur and proximal tibia^1^. Owing to its high degree of malignancy and rapid metastasis, OS prognosis remains poor with relatively low survival rates. Currently, the standard treatment for OS includes surgical resection combined with preoperative and postoperative adjuvant radiotherapies (high-dose methotrexate, azithromycin, and cisplatin). Although this strategy has made a significant contribution to improving the overall survival rates in these patients, the metastasis, invasion, and recurrence rates of OS remain high, and patients who are prone to lung metastases often die. In addition, the use of a large number of chemotherapy drugs not only increases the risk of adverse reactions but also aggravates tumor resistance, seriously affecting the quality of life of patients and complicating OS treatment. Therefore, a safe and effective drug should be developed to help reduce OS cell invasion and increase its chemosensitivity^2^.

Herbs contain safe natural compounds with few side effects, are inexpensive and easily accessible and have a positive adjunctive effect in the treatment of various conditions, including OS. *Paris polyphylla*, *paclitaxel*, *Curcuma longa*, *Tripterygium wilfordii*, and other traditional Chinese medicines (TCMs) can promote apoptosis and autophagy in OS cells and even increase their sensitivity to some chemotherapy drugs, thus making TCMs effective in treating OS^3^. The TCM theory is that OS is caused by a deficiency in healthy qi and the invasion of evil qi, causing phlegm coagulation, blood stasis, asthenia, toxins, and other pathological factors associated with OS. The principles of TCM treatment include strengthening the healthy qi and expelling evil qi by tonifying the qi and blood, regulating the viscera, detoxification and removal of blood stasis, among others^3^. Curcuma is a dried rhizome of the ginger plant curcuma that causes blood thinning, promotes qi, and relieves pain. Curcuma is often used as a TCM to treat chest pain, abdominal masses, wind‒cold‒dampness arthralgia, and other diseases. Modern pharmacological studies have shown that many compounds of the TCM curcuma have anticancer activity; curcumin in particular has a significant antitumor effect. Curcumin inhibits the proliferation of OS cells and induces their apoptosis through various metabolic pathways. Noncurcumin compounds in curcuma, such as aromatic curcumone, curcumol, and curdione, have also been shown to have anticancer activity^4,5^. However, the precise mechanism underlying the anticancer activity of curcumin and other compounds in curcuma in OS remains unclear. The potentially complex molecular mechanisms of active antitumor compounds in curcuma that can be used for OS treatment require further study.

**Figure 1:**
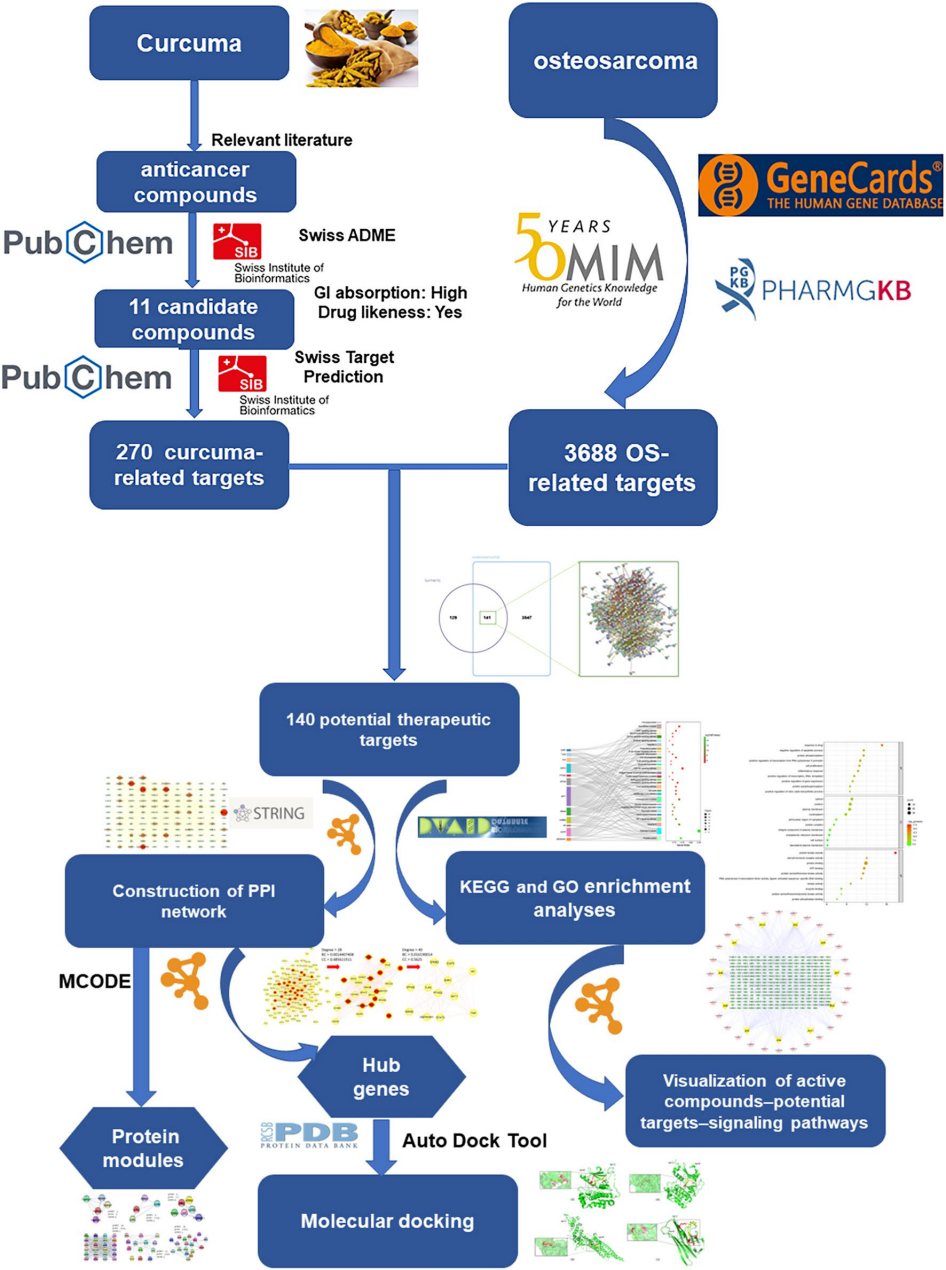
A flow-chart of this study to explore the potential mechanism of curcuma in the treatment of OS.

Network pharmacology, a combination of pharmacology and pharmacodynamics, is a novel research field that allows investigators to clarify the synergistic effects and underlying mechanisms of numerous compounds by facilitating the evaluation of their underlying multilevel interactions. Therefore, this study aimed to determine the therapeutic targets and potential signaling pathways most closely related to OS to determine the potential mechanism of curcuma as an anticancer agent in this disease. The protocol of our study procedures is provided in Fig. 1.

## Materials and methods

### Chemical candidates and curcuma-related targets

All curcuma compounds with anticancer activity were obtained from relevant literature. The chemical candidates were then screened using the Swiss ADME database (http://www.swissadme.ch/) (accessed on 6th December 2022)^6^ based on two key ADME indices: GI absorption and drug likeness. The GI absorption index indicates the ability of the drug to be digested and absorbed in the stomach and intestines after oral administration. The drug likeness index assesses the likelihood of a molecule becoming an oral drug with respect to bioavailability. Therefore, only compounds with high “Gi absorption” and favorable “drug-likeness” were considered candidates. Subsequently, the active candidate compounds were inputted into PubChem (https://pubchem.ncbi.nlm.nih.gov/) (accessed on 6th December 2022)^7^ to collect the chemical structures using the Simplified Molecular Input Line Entry System. Curcuma-related targets with a probability greater than 0 were predicted using the Swiss Target Prediction database (http://www.swisstargetprediction.ch/) (accessed on 6th December 2022)^8^.

### Identification of OS-related targets

We searched for OS-associated targets in three databases, including the GeneCards database (https://www.genecards.org/) (accessed on 8th December 2022)^9^, which provides comprehensive, user-friendly information on all annotated and predicted human genes and automatically integrates gene-centric data (e.g. genomic, transcriptomic, proteomic, genetic, clinical, and functional information) from ~ 150 web sources; the OMIM database (https://omim.org/) (accessed on 8th December 2022)^10^, which is a comprehensive, authoritative compendium of human genes and genetic phenotypes that is freely available and updated daily and contains information on all known Mendelian disorders, over 16,000 genes; and the PharmGKB database (https://www.pharmgkb.org/) (accessed on 8th December 2022)^11^. Finally, the targets from each database were merged and labeled as OS-related targets.

### Construction and analysis of the protein‒protein interaction (PPI) network

STRING (https://string-db.org/) (accessed on 8th December 2022)^12^ is a database that integrates all known and predicted associations between proteins, including both physical and functional interactions. In this study, we used the STRING database to analyze the relationship of common curcuma and OS-related targets, which were considered potential therapeutic targets. The PPI network of potential therapeutic targets was constructed using the following conditions: the species was "*Homo sapiens*", and the reliability was "medium confidence of ≥ 0.4". A confidence score of 0.4 is often used to indicate medium confidence when predicting protein‒protein interactions. This particular value was selected due to its ability to balance sensitivity and specificity while also serving as a dependable threshold for identifying potential interactions. Therefore, only proteins with interaction relationship scores greater than or equal to 0.4 are included in the protein network. Subsequently, the PPI network was analyzed using Cytoscape v3.8.0 software to obtain topology parameters of nodes in the network, including degree value, betweenness centrality (BC) value, and closeness centrality (CC) value. The “degree” indicates the number of nodes in the network that act directly with the node and is commonly used to assess node importance in networks. Analogous to the former, BC and CC are measures of a node's importance in a network based on its ability to interact with other nodes in the network or act as a bridge for information flow between other nodes. Therefore, the hub genes of the PPI network were selected based on the cutoff criteria: first, genes with a degree value (28), BC value (0.00144074408), and CC value (0.485611511) greater than the median value in the entire set were screened out; then, the genes with degree (40), BC (0.016190014), and CC (0.5625) values greater than the median in this gene set were screened out. The MCODE plugin was used for module clustering of the PPI network.

### Functional enrichment and pathway analysis

All potential therapeutic targets were subjected to Gene Ontology (GO) and Kyoto Encyclopedia of Genes and Genomes (KEGG) pathway enrichment analysis via the DAVID database (https://david.ncifcrf.gov/) (accessed on 10th December 2022)^13^ to identify the related pathways and related GO terms, including those in the biological process (BP), molecular function (MF), and cellular component (CC) categories. The pathways and GO terms with applicable thresholds of p < 0.05 were considered significant and were retained. Additionally, Bioinformatics (http://www.bioinformatics.com.cn/) (accessed on 13th December 2022) was used to visualize the GO and KEGG enrichment analysis results in a bar graph of signaling pathways and a bubble plot of GO categories. The network of active compounds‒potential targets‒signaling pathways was visualized using Cytoscape v3.8 software.

### Verification with molecular docking

To analyze the relationship between the key targets of curcuma and the core compound, a molecular docking approach was employed. The 3D structure of the core compound was obtained from the PubChem database and imported into Chem3D software for energy minimization, resulting in the pdb file of small molecules. The 3D structures of the core target were downloaded from the PDB database (http://www.rcsb.org/) (accessed on 11th December 2022)^14^, and we selected the protein 3D structure with high quality based on the following criteria: Source organism is “Homo sapiens”, Refinement resolution within 2.5A, and complete protein structure sequence with information of small molecule ligands. Subsequently, water molecules and the original ligand of the proteins obtained were removed using PyMOL software. AutoDock Tool 1.5.7 software was used to hydrogenate, calculate charge and combine nonpolar hydrogen, and the ligands and receptors were stored as PDBQT files. All the flexible bonds of the small molecule ligand were set to be rotatable. Consequently, the Lamarckian genetic algorithm was used with a maximum eval number as the medium, and the Ga Crossover mode “twopt” was used to perform rigid docking and identify the binding activity of ligand and receptors. Finally, the reliability of the docking results was assessed by calculating the root mean square deviation (RMSD) value using PyMOL software.

## Results

### Compounds and curcuma-related targets

Curcuma compounds with anticancer activity were obtained by searching the relevant literature^5,14^. Using the SwissADME tool, we screened eleven active curcuma compounds with high gastrointestinal (GI) absorption and drug-like properties (Table 1). These compounds included curcumin, curcumol, Ar-turmerone, alpha-turmerone, beta-turmerone, curdione, cyclocurcumin, germacrone, furanodiene, calebin A, and furanodienone, which may be the material basis for curcumin's anti-osteosarcoma (OS) effect. *β-Sesquiphellandrene*, *δ-elemene*, and *δ-elemene* were excluded due to their low GI absorption. In addition, the potential targets of each candidate component were predicted using the SwissTarget Prediction database (Supplementary Table S1). We then removed any duplicate targets and merged the remaining targets to obtain a total of 270 active compound-related targets.

**Table 1:** ADME information of anticancer compounds in curcuma.

Compound	GI absorption	Druglikeness	Water solubility	Molecular weight
Curcumin	High	YES	Soluble	368.38
Ar-turmerone	High	YES	Soluble	216.32
β-sesquiphellandrene	Low	YES	Moderately soluble	204.35
Alpha-turmerone	High	YES	Soluble	218.33
β-elemene	Low	YES	Moderately soluble	204.35
Beta-turmerone	High	YES	Soluble	218.33
δ-Elemene	Low	YES	Moderately soluble	204.35
Curdione	High	YES	Soluble	236.35
Cyclocurcumin	High	YES	Moderately soluble	368.38
Germacrone	High	YES	Soluble	218.33
Furanodiene	High	YES	Moderately soluble	216.32
Curcumol	High	YES	Soluble	236.35
Calebin A	High	YES	Moderately soluble	384.38
Furanodienone	High	YES	Moderately soluble	230.3

### Potential therapeutic targets of curcuma used in the treatment of OS

**Figure 2:**
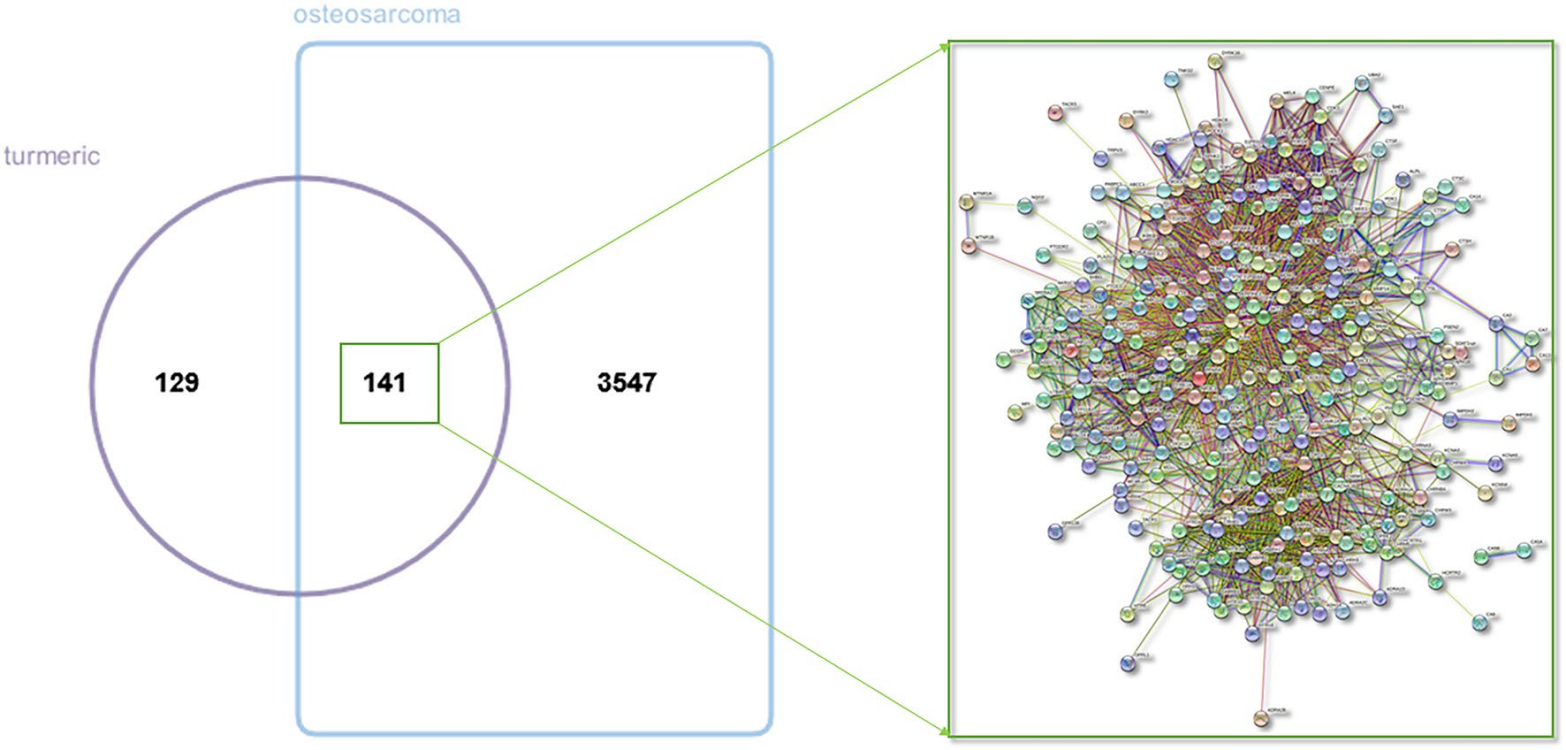
Venn diagram of curcuma and OS-related targets.

Data on “osteosarcoma,” which was used as the keyword, were retrieved from various databases. A total of 3688 targets were searched, including 3655 from GeneCards, 11 from OMIM, and 97 from PharmGKB (Supplementary Table S2). Of the curcuma- and OS-related targets, 141 common targets were identified and considered potential therapeutic targets of curcuma used in the treatment of OS, as shown in the Venn diagram (Fig. 2).

### PPI network visualization and analysis

**Figure 3:**
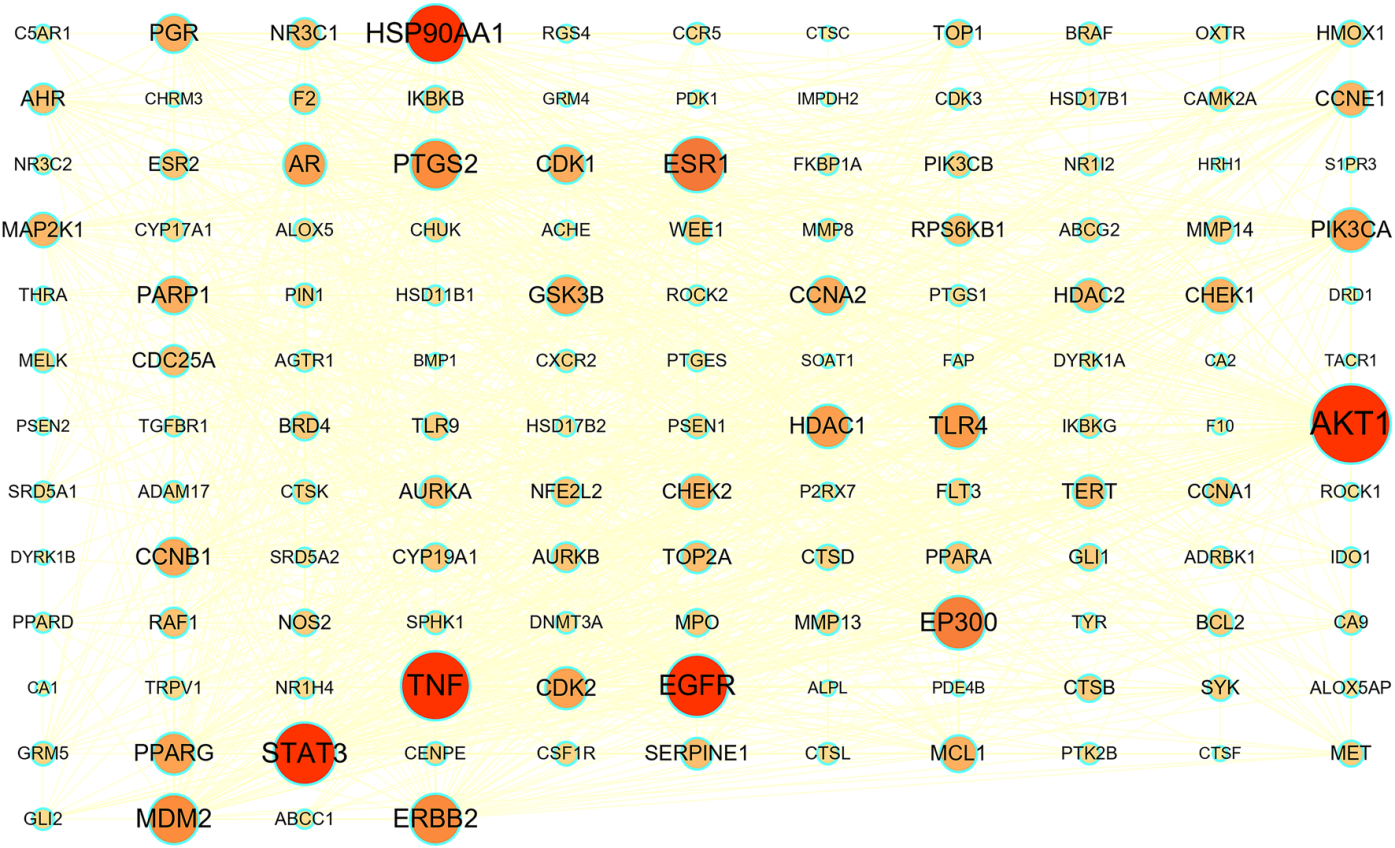
PPI network: the darker the color, the larger the node, the larger its degree value in the network, and the greater its importance within the network.

**Figure 4:**
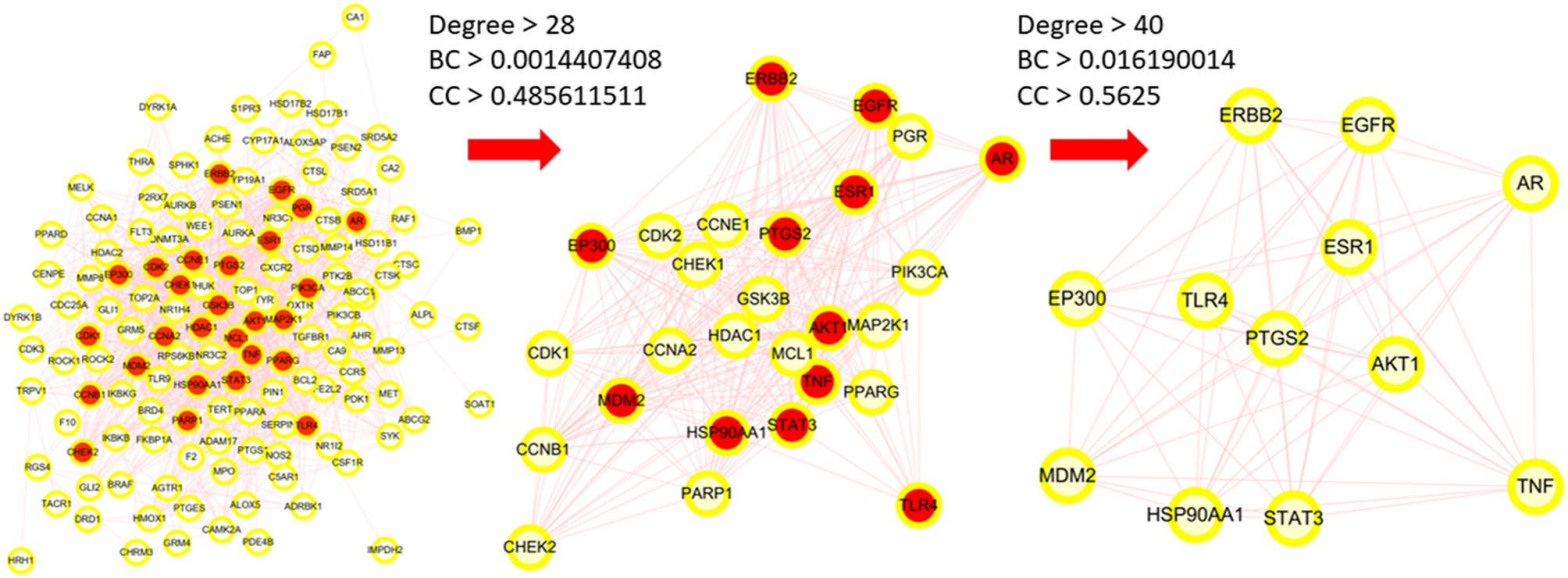
PPI network hub gene screening process: The red nodes represent the targets that meet the screening conditions.

**Figure 5:**
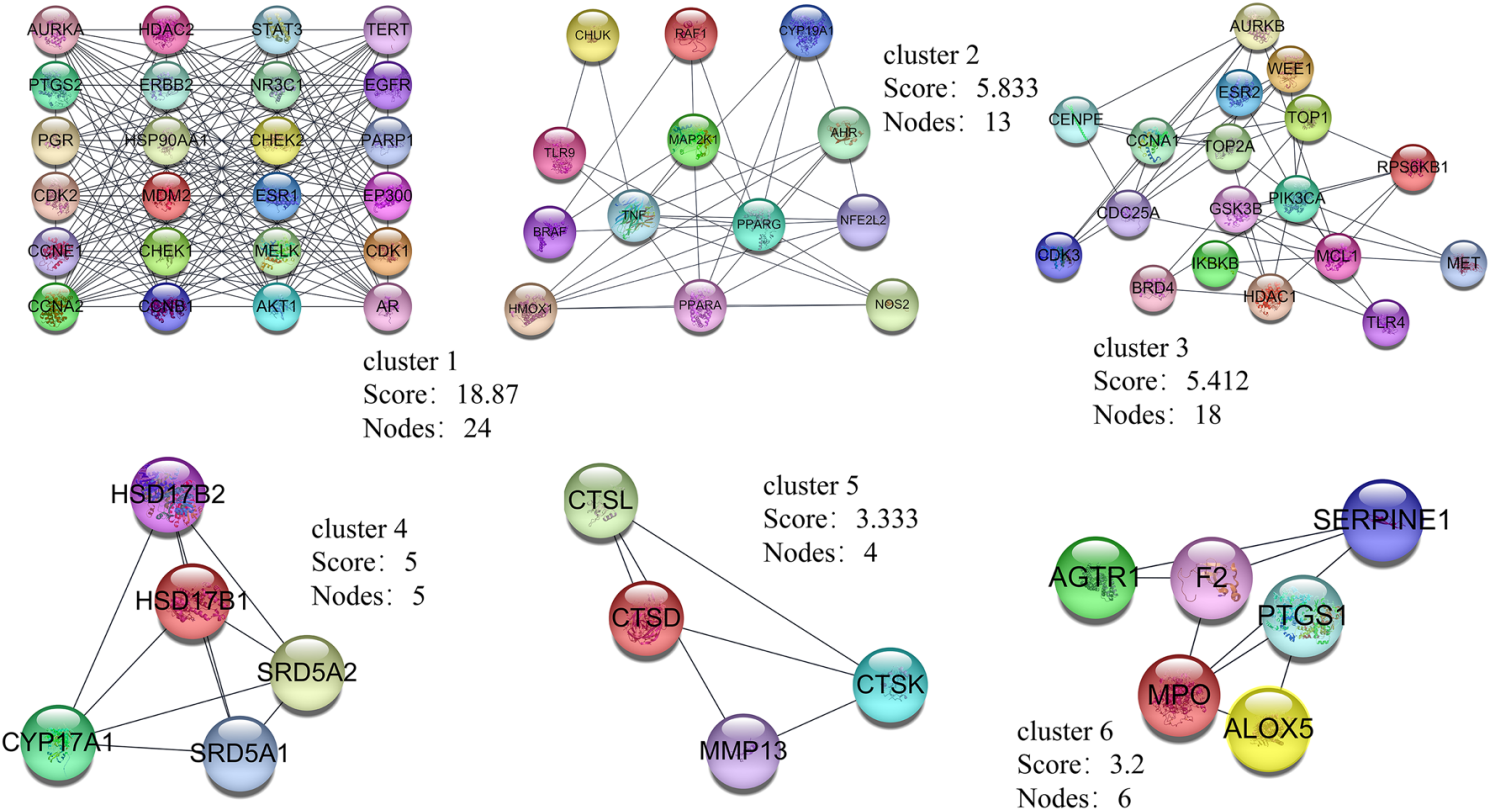
Cluster analysis of the protein modules within the PPI network.

The STRING database was used to analyze the relationship between 141 potential therapeutic targets (Supplementary Table S3). Cytoscape v3.8.0 software was used for the visualization of the PPI network. According to the visualization, the PPI network included 141 nodes and 1279 edges, and the average node degree was 18.1 (Fig. 3). The nodes represent the individual proteins, while the edges represent the connections between the proteins. The greater the degree value, the more significant the role of the protein in the network. After filtering using the above criteria (Fig. 4), 12 hub genes were identified: *AKT1*, *TNF*, *STAT3*, *EGFR*, *HSP90AA1*, *ESR1*, *EP300*, *ERBB2*, *PTGS2*, *MDM2*, *TLR4*, and *AR* (Table 2). These genes have superior network topology parameters, such as degree, BC, and CC, and thus are considered core targets in the PPI networks. These proteins are biological enzymes and cytokines, which are involved in various biological regulation processes, such as human signal transcription and protein phosphorylation. *AKT1*, *TNF*, *STAT3*, *EGFR*, and *HSP90AA* were the top five nodal targets; they showed a strong association with other potential therapeutic targets and play an important role in the treatment of OS. The MCODE plugin was used for the cluster analysis of protein groups, and six cluster modules (Fig. 5) were obtained. Twelve important protein targets, including *AKT1*, *TNF*, *STAT3*, and *EGFR*, were clustered in the top two functional modules. *AKT1* and *TNF* were identified as the core nodes in each of the two functional modules, suggesting that these two key targets significantly influence the effects of curcuma against OS.

**Table 2:** Descriptions of the PPI network hub genes.

Target	Degree	Closeness centrality	Betweenness centrality	Type
AKT1	90	0.75	0.169699718	Protein kinase
TNF	76	0.68877551	0.123520888	Cytokines
STAT3	67	0.645933014	0.050619783	Cytokines
EGFR	67	0.658536585	0.071173123	Signaling molecule
HSP90AA1	62	0.627906977	0.051528057	Molecular chaperone
ESR1	57	0.619266055	0.053214005	Signaling molecule
EP300	55	0.610859729	0.037532744	Transcriptase
ERBB2	51	0.602678571	0.029911196	Protein kinase
PTGS2	50	0.597345133	0.031902439	Regulatory enzyme
MDM2	50	0.589519651	0.020425553	Cytokines
TLR4	43	0.5625	0.02032193	Signaling molecule
AR	40	0.564853556	0.022699694	Signaling molecule

### GO enrichment analyses

**Figure 6:**
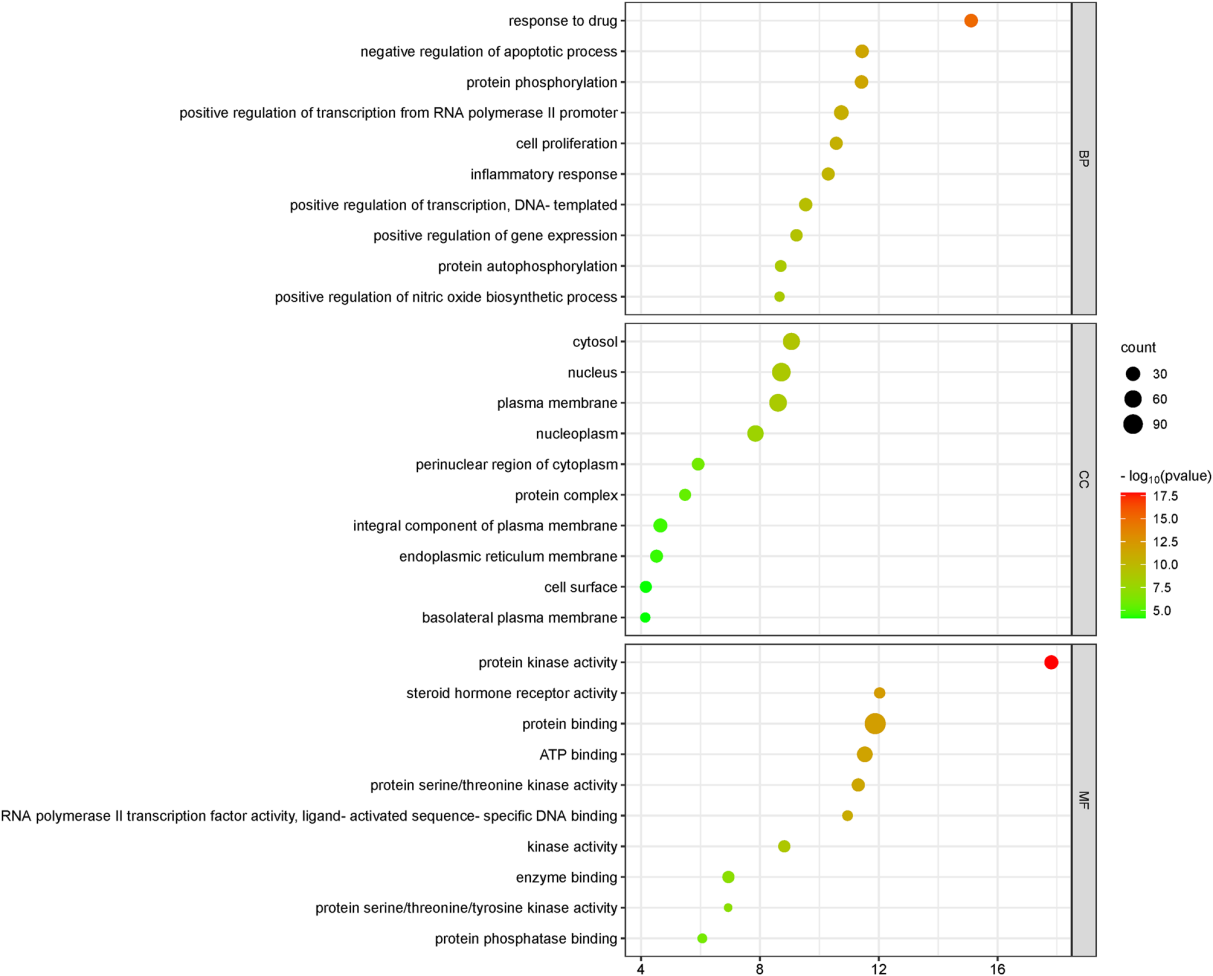
Bubble plot of the top 10 BP, MF and CC categories associated with curcuma and OS.

To determine the biological mechanisms of curcuma against OS, we performed GO enrichment analysis on 141 potential therapeutic targets of curcuma used in the treatment of OS using the DAVID database. The analysis was divided into three categories: BP, MF, and CC. We obtained 295 BP, 80 MF, and 42 CC terms, and the top 10 GO terms were visualized in the bubble plot (Fig. 6). The bubble size and color indicate the enrichment gene and p value, respectively. A darker color indicates a smaller p value, a larger bubble, and a higher number of enriched therapeutic genes within that GO item, indicating that the identified GO term is more strongly associated with the treatment of OS than other GO terms. Our analysis revealed that the main enriched BP categories were the response to drugs, positive regulation of transcription from RNA polymerase II promoter, negative regulation of apoptotic process, protein phosphorylation, cell proliferation, and inflammatory response. The main enriched MFs were protein binding, ATP binding, and protein kinase activity. The core targets, such as protein kinases, transcriptases, signaling molecules, and cytokines, were involved in biological metabolism, as supported by the BP and MF results. CC analysis revealed that the nucleus, plasma membrane, cytosol, and nucleoplasm were the most enriched CC terms. The inflammatory response, cell proliferation, RNA transcription energy conversion, and protein binding frequently take place in these intracellular sites.

### KEGG pathway enrichment analyses

**Figure 7:**
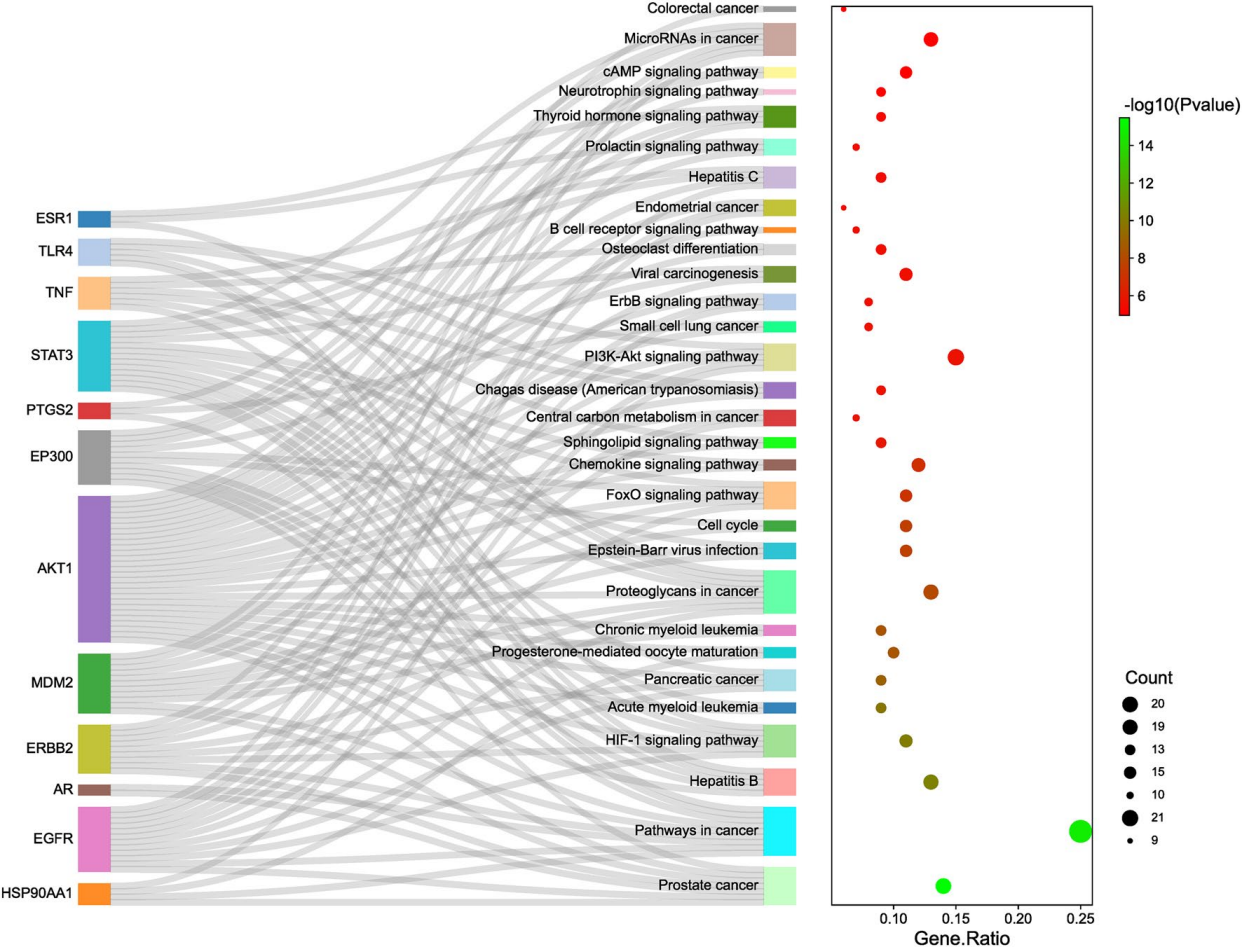
Bar graph of the top 30 signaling pathways.

**Figure 8:**
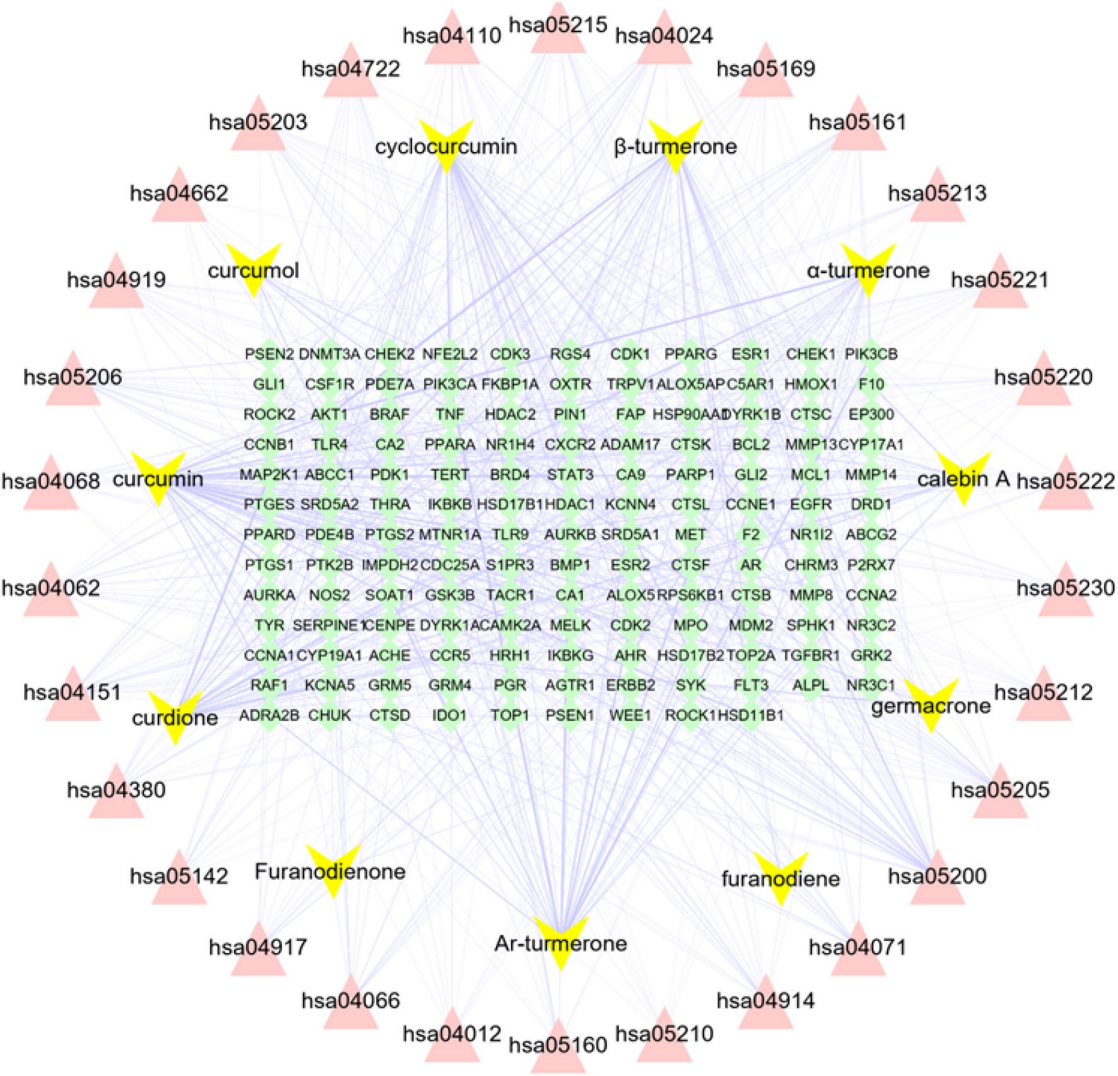
Visualization of active compounds‒potential targets‒signaling pathways: yellow nodes represent the active compound, pink nodes represent the signaling pathway, and green nodes represent the potential target. hsa05215-Prostate cancer; hsa05200-Pathways in cancer; hsa05161-Hepatitis B;hsa04066-HIF-1 signaling pathway; hsa05221-Acute myeloid leukemia; hsa05212-Pancreatic cancer; hsa04914-Progesterone-mediated oocyte maturation; hsa05220-Chronic myeloid leukemia; hsa05205-Proteoglycans in cancer; hsa05169-Epstein-Barr virus infection; hsa04110-Cell cycle; hsa04068-FOXO signaling pathway; hsa04062-Chemokine signaling pathway; hsa04071-Sphingolipid signaling pathway; hsa05230-Central carbon metabolism in cancer; hsa05142-Chagas disease (American trypanosomiasis); hsa04151-PI3K-Akt signaling pathway; hsa05222-Small cell lung cancer; hsa04012-ErbB signaling pathway; hsa05203-Viral carcinogenesis; hsa04380-Osteoclast differentiation; hsa04662-B cell receptor signaling pathway; hsa05213-Endometrial cancer; hsa05160-Hepatitis C; hsa04917-Prolactin signaling pathway; hsa04919-Thyroid hormone signaling pathway; hsa04722-Neurotrophin signaling pathway; hsa04024-cAMP signaling pathway; hsa05206-MicroRNAs in cancer; hsa05210Colorectal cancer.

The metabolic pathways of potential therapeutic targets for the treatment of OS were identified through KEGG pathway enrichment analyses. Using the DAVID database, we obtained 87 signaling pathways. We plotted the top 30 signaling pathways in a bar graph (Fig. 7), sorted by their P values from smallest to largest (Supplementary Table S4). The results showed that the key targets were enriched in the HIF-1 signaling pathways, PI3K-Akt signaling pathways, and chemokine signaling pathways. To further analyze the relationship between active compounds, potential therapeutic targets, and critical pathways, we constructed a visual network using Cytoscape v3.8.2 (Fig. 8). The visual network comprised 182 nodes and 707 edges. Curcumin, which was closely connected with 48 potential therapeutic targets and had the highest degree value (degree = 48) among candidate compounds, showed a strong association with several core OS targets (AKT1, EGFR, EP300, and STAT3), and was considered the most important compound. Therefore, we selected it as the key compound for molecular docking with key targets, including AKT1, TNF, STAT3, and EGFR, via Auto Dock Tool4.2 software.

### Molecular docking

**Figure 9:**
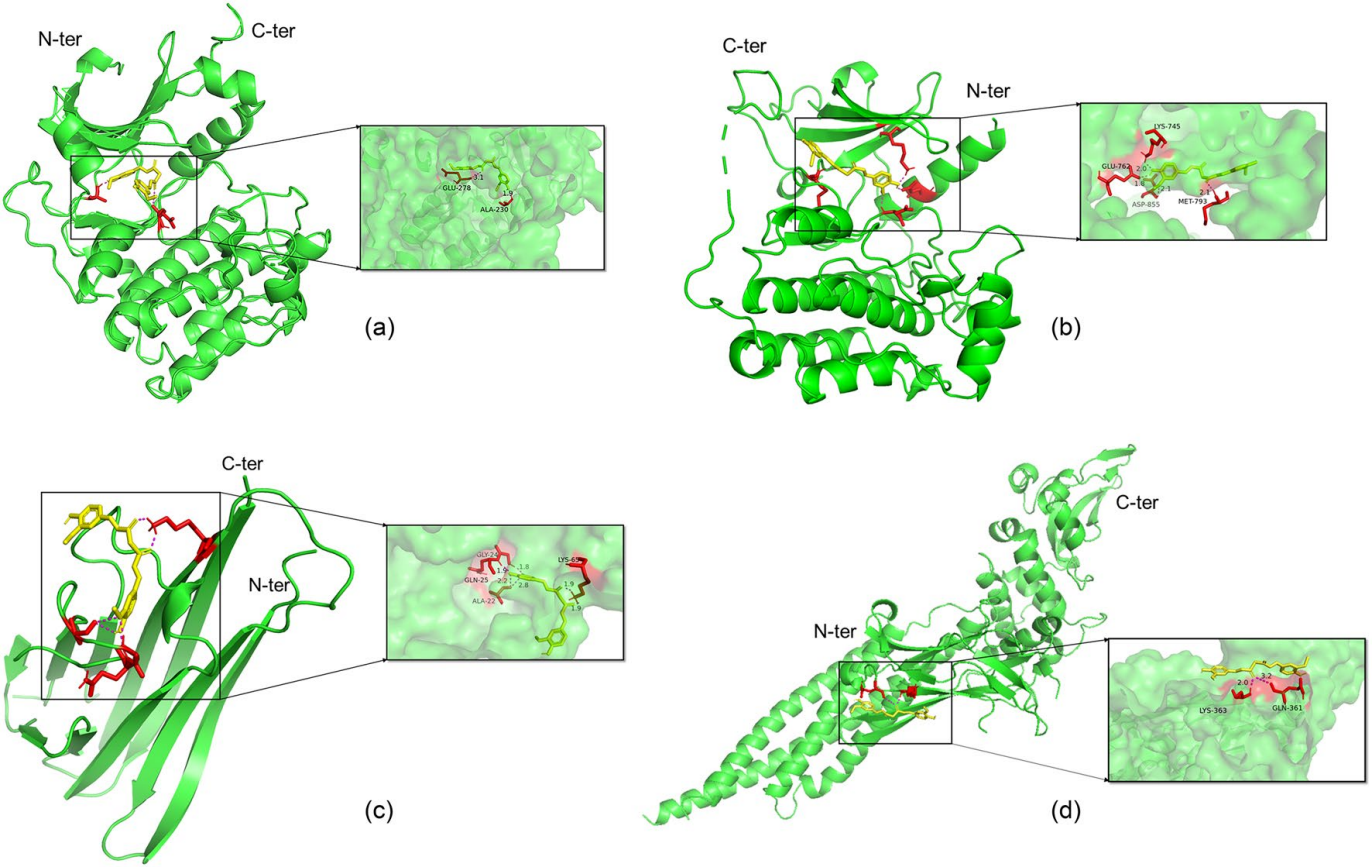
(**a**) Molecular docking model of curcumin with AKT1. (**b**) Molecular docking model of curcumin with TNF. (**c**) Molecular docking model of curcumin with EGFR. (**d**) Molecular docking model of curcumin with STAT3.

When the conformation of the ligand and receptor is stable, the possibility of action increases as the binding energy decreases. A binding energy of less than 0 kJ/mol indicates spontaneous binding between the ligand and receptor, while a binding energy of less than − 5 kJ/mol suggests that the ligand and receptor bind closely. A lower RMSD value indicates more accurate docking results, and a value of less than 2A confirms the validity of the docking result. The molecular docking results showed that curcumin had a strong affinity for AKT1, TNF, STAT3, and EGFR, with low RMSD values indicating reliable results. The minimum binding energies and RMSD values are listed in Table 3. Additionally, curcumin interacted with specific residues by forming hydrogen bonds between residues including GLU278 and ALA230 of AKT1; GLY24, GLN25, ALA22, and LYS65 of TNF; GLN361 and LYS363 of STAT3; and GLU762, LYS745, ASP855, and MET793 (Fig. 9).

**Table 3:** Molecular docking model information and docking results.

Targets	PDB ID	Original ligand	Active pocket (X,Y,Z)	Binding energy (kJ/mol)	RMSD/A
AKT1	3CQU	CQU	1.254, − 0.77, 25.125	− 30.7942	0.876
TNF	2AZ5	307	− 26.571, 65.941, 41.94	− 26.23368	1.601
STAT3	6NJS	KQV	− 6.714, 19.467, 24.491	− 22.76096	1.628
EGFR	6LUD	YY3	− 57.746, − 8.003, − 24.711	− 26.9868	1.784

## Discussion

OS is a primary malignancy associated with malignant mesenchymal cells with a high propensity for local invasion and early metastasis. Currently, the standard treatment for OS is surgery combined with chemotherapy. In recent years, the treatment of OS has progressed rapidly, and many treatment modalities, such as neoadjuvant chemotherapy combined with limb-sparing surgery, radiation, gene therapy, immunotherapy, and TCM, have emerged and have greatly improved the survival rate of patients with OS within 5 years^15^. TCM has long been used in the treatment of OS, and its mechanism is highly complex, involving multiple targets and pathways, as demonstrated by several studies. For example, Zhao et al. reported that Polygonum cuspidatum inhibits Akt/ERK/EGFR pathways to exert its effects on the cell cycle and apoptosis of OS cells^16^. Zhang et al. have also shown that Angelica dahurica targets multiple pathways, including the MAPK signaling pathway, PI3K-Akt signaling pathway, and TNF signaling pathway, to treat OS by acting on multiple targets, such as ESR and TNF^17^. Curcuma is also a commonly applied TCM with anti-inflammatory, anticoagulant, analgesic, antitumor, hypolipidemic, and other pharmacological effects. Clinically, the antitumor effect of curcuma is particularly prominent. However, the precise mechanism underlying its anticancer activity against OS remains unclear.

In the present study, we utilized network pharmacology to predict the mechanism underlying the therapeutic effects of curcuma for treating OS. We identified and screened 11 active antitumor compounds, including curcumin, curcumol, and calebin A, which serve as the material basis of anti-OS activities. In clinical practice, these compounds have been associated with apoptosis and drug resistance in tumor cells. Specifically, curcumin, curcumol, and calebin A exert anti-OS effects through multiple mechanisms. For example, curcumin is the main active compound of curcuma and can exert its antitumor effect by inhibiting tumor cell proliferation, regulating the energy metabolism of tumor cells, inhibiting tumor invasion and local angiogenesis, inducing tumor cell apoptosis and autophagy, inhibiting tumor immune escape, and reversing tumor cell resistance^18^. In a randomized controlled study of MG-63 cisplatin-resistant OS cell lines, Chen et al. found that curcumin inhibits autophagy activation in MG-63 cisplatin-resistant OS cells and promotes apoptosis in cisplatin-induced OS-resistant cells^19^. In addition, curcuminol, a noncurcumin compound of curcuma, inhibits the proliferation of OS cells by inducing autophagy^20^. Calebin A, an anticancer-active compound isolated from curcuma, inhibits osteoclasts induced by tumor cells by inhibiting the nuclear factor kappa B (NF-κB) pathway^21^.

We predicted anticancer-active compounds and OS-related targets in curcuma from a public database and found that curcuma has 141 interrelated common targets against OS, which induce cell apoptosis and proliferation. By analyzing the interactions between potential therapeutic targets, we found that these targets are clustered in these six protein modules. that have various biological functions. For example, module one is related to the G2/M transition of the mitotic cell cycle, protein phosphorylation, and regulation of signal transduction by p53 class mediator. Moreover, module two is related to positive regulation of transcription from the RNA polymerase II promoter, response to hypoxia, positive regulation of transcription, and DNA templating. Moreover, these 141 potential therapeutic targets are closely related to other targets; therefore, they are considered “hubs” in the therapeutic network. These 12 core proteins play a synergistic role involving the molecular mechanisms of proliferation and apoptosis, angiogenesis, metastasis, invasion, and drug resistance of OS cells. In particular, *AKT1*, *TNF*, *STAT3*, *EGFR*, *HSP90AA1*, and *PTGS2* play important roles in the pathogenesis of OS. *AKT1* (RAC-alpha serine/threonine-protein kinase) interacts with the NF-κB activation receptor, which is a critical signaling pathway in bone tumors and osteogenesis. Its inhibition may suppress the proliferation of OS cells^22^. Liu et al. found that *AKT1* gene knockout significantly inhibited the migration and proliferation of OS cells, highlighting its importance as an oncogene in OS cell growth and differentiation^23^. TNF*-α* is also associated with OS, and several related clinical studies have shown that the serum TNF-α level in patients with OS is significantly higher than that in healthy individuals^24,25^. Additionally, TNF-α is related to the pulmonary metastasis of OS. In vitro and in vivo studies have shown that TNF-α inhibitors reduce the expression of chemokine receptors and effectors to decrease cellular activity and pulmonary metastasis in OS cells, thus preventing pulmonary metastasis of OS^26^. STAT3 is another signaling and transcriptional activator involved in cell differentiation, growth, and survival with various biological functions, including embryogenesis, immunity, hematopoiesis, and cell migration. As a proto-oncogene, STAT3 is commonly overexpressed in OS tissues and cell lines, resulting in the decreased expression of the tumor suppressor gene *PARK2*^27^. The use of STAT3 inhibitors blocks downstream signaling and protein synthesis in OS cells and plays an active role in the treatment of OS^28^. *EGFR* is involved in the proliferation, differentiation, and migration of OS cells, and its expression increases the resistance of OS cells to certain anticancer drugs^29^. HSP90AA1 is a molecular chaperone known to function as an essential regulator of autophagy in OS cells and promotes autophagy through the PI3K/Akt/mTOR and JNK/P38 signaling pathways, inhibiting apoptosis and increasing chemotherapeutic tolerance in these cells^30^. This particular response is also thought to be a key mechanism of drug resistance in OS cells. Consequently, curcuma might act on these targets to treat OS.

GO analysis provides an in-depth definition and description of the functions of potential therapeutic target proteins and genes, reflecting the potential mechanism of curcuma in combatting OS treatment resistance. Some important BPs, such as the regulation of apoptosis, cell proliferation, and inflammatory response, are associated with these potential therapeutic targets. In the OS microenvironment, bone, stromal, vascular, and immune cells, along with the mineralized extracellular stroma, coexist and coordinate to support various physiological processes, such as bone lysis, vascular reconstruction, and immunomodulation, which are associated with OS progression. In addition, the inflammatory response is closely related to the occurrence and progression of bone cancer, and several of the key targets identified in this study, including cytokines (such as TNF and PTGS2) and inflammatory reaction enzymes, are involved in regulating the inflammatory microenvironment of OS^31,32^. This environment depends on the activity of various protein kinases, steroid hormone receptors, RNA polymerase II transcription factors, kinases, and MFs, such as protein, enzyme, protein kinase, and energy substance binding. Moreover, these functions and BPs are also associated with specific cellular substructures, such as the nucleus, plasma membrane, cytosol, and nucleoplasm.

Our KEGG enrichment analysis identified multiple signaling pathways involved in the anti-OS mechanism of curcuma. The core gene targets were enriched in the PI3K/Akt, HIF-1, and FOXO signaling pathways, indicating that these signaling pathways are extremely important. By intervening in these signaling pathways, the BPs in which the core targets are involved can be mostly mediated. Moreover, these signaling pathways are closely related to the development and metastasis of OS. Therefore, curcuma may play an anti-OS role by interfering with tumor cell proliferation, angiogenesis, metastasis, invasion, and chemotherapy resistance. The PI3K/AKT signaling pathway is an important carcinogenic pathway in human cancers, including OS. It is involved in multiple pathological processes, such as tumorigenesis, proliferation, invasion, cell cycle progression, apoptosis, angiogenesis, metastasis, and chemotherapy resistance in OS. In this pathway, AKT phosphorylation significantly upregulates the expression of the oncogene c-Myc and promotes the proliferation and migration of OS cells. It also inhibits the programmed death of OS cells, which is closely related to OS tissue growth. Additionally, this pathway is associated with lung metastasis in patients with OS^33^. The HIF-1 signaling pathway is closely related to the migration and invasion of OS cells. It is involved in the angiogenesis of OS tissues and promotes the proliferation of OS cells. HIF-1, as a biomarker of OS, activates the AKT/cyclin D1 signaling cascade to promote the growth, migration, and invasion of OS cells in vivo. In a mouse model, overexpression of HIF-1 promoted the growth of tumor blood vessels and the growth and development of LM8 OS cells^34,35^. FOXOs are known tumor suppressors that promote tumor cell apoptosis and inhibit cell proliferation. In the metabolic environment of OS, FOXOs reduce osteoblast generation by inhibiting the classical Wnt/β-catenin signaling pathway^36^. The chemokine signaling pathway plays a key role in tumorigenesis and development. The CXCL12/CXCR4 axis activates multiple pathways involved in angiogenesis, metastasis, and survival in the tumor microenvironment and promotes tumor cell invasion and metastasis^37^. The CCL5/CCR5 axis promotes OS cell migration and supports angiogenesis^38^, while the ErbB signaling pathway is associated with OS pathogenesis. Cruz-Ramos et al. found that the application of EGFR blockers reduced the activity of OS cells, decreasing the probability of metastasis^39^. Moreover, Wang found that EGFR is a therapeutic target for inhibiting the invasion of OS, which is related to the unfavorable prognosis of OS. In the ErbB signaling pathway, EGF and its receptor activate MMP9, a protease involved in the proliferation of tumors, resulting in the invasion of cancer cells^40^. The thyroid hormone signaling pathway is of great significance in osteogenesis*. *In vitro studies by Liu et al. revealed that overexpression of thyroid hormone receptor-interacting proteins promoted the proliferation and migration of OS cells^41^.

Overall, the results based on network pharmacology and molecular docking presented in this study provide important insights into the potential anticancer mechanisms of curcuma by highlighting its ability to interact with multiple targets involved in cancer development and progression. Despite these findings, there are still some limitations to this study. The development of disease involves a pathological process. The curative effect of the compound differs significantly at each stage, suggesting that OS stage should be considered in treatment selection. The therapeutic effects of different doses of curcuma on the treatment of OS should be considered. Additionally, since the study was based on database analysis and computational approaches, there is a lack of experimental findings to validate the results of our research. Therefore, further experimental studies on these pathways and targets are necessary to validate the mechanisms of action.

## Conclusion

Our research, based on network pharmacology and molecular docking, revealed that curcuma-mediated treatment of OS was a complex process involving multiple compounds, targets, and pathways. Through the PI3K-Akt, HIF-1, and ErbB signaling pathways, curcuma regulates key targets that are involved in angiogenesis, cancer cell proliferation, metastasis, invasion, and chemotherapy resistance, including *AKT1*, *TNF*, *STAT3*, *EGFR* and HSP90AA1, in the treatment of OS. These targets and pathways not only provide a pharmacological basis for the clinical treatment of OS but also provide a theoretical basis for the development of new drugs that interfere with lung metastasis and drug resistance in OS.

## Supplementary Information

Supplementary Tables.

## Data Availability

The datasets of the current study are available in public database from SwissADME database (http://www.swissadme.ch/), PubChem (https://pubchem.ncbi.nlm.nih.gov/), Swiss Target Prediction (http://www.swisstargetprediction.ch/), GeneCards (https://www.genecards.org/), OMIM (https://www.omim.org/), PharmGkb (https://www.pharmgkb.org), STRING (https://string-db.org/), DAVID database (https://david.ncifcrf.gov/) and PDB (https://www.rcsb.org/). Supplementary files are provided for compound targets, disease targets, intersection targets, and 30 selected KEGG information. The data used to support the findings of this study are available from the corresponding author upon reasonable request.

## Abbreviations

OS: Osteosarcoma
GO: Gene ontology
KEGG: Kyoto encyclopedia of genes and genomes
TCM: Traditional Chinese medicine
ADME: Absorption, distribution, metabolism, and excretion
BC: Betweenness centrality
CC: Closeness centrality
BP: Biological process
MF: Molecular function
PPI: Protein–protein interaction
*AKT1*: RAC-alpha serine/threonine-protein kinase
TNF: Tumor necrosis factor
STAT3: Signal transducer and activator of transcription 3
EGFR: Epidermal growth factor receptor
HSP90AA1: Heat shock protein HSP 90-alpha
ESR1: Estrogen receptor
EP300: Histone acetyltransferase p300
ERBB2: Receptor tyrosine-protein kinase erbB-2
PTGS2: Prostaglandin-endoperoxide synthase2
MDM2: E3 ubiquitin-protein ligase Mdm2
TLR4: Toll-like receptor 4
AR: Androgen receptor
